# Pigmentary Disorders: Elucidation of Pathogenesis for Recovery of Health and Wellness

**DOI:** 10.1111/1346-8138.70157

**Published:** 2026-01-27

**Authors:** Naoki Oiso

**Affiliations:** ^1^ Department of Dermatology Kindai University Nara Hospital Ikoma Japan

**Keywords:** chemical‐induced vitiligo, chemical leukoderma, genetic pigmentary disorders, vitiligo

1

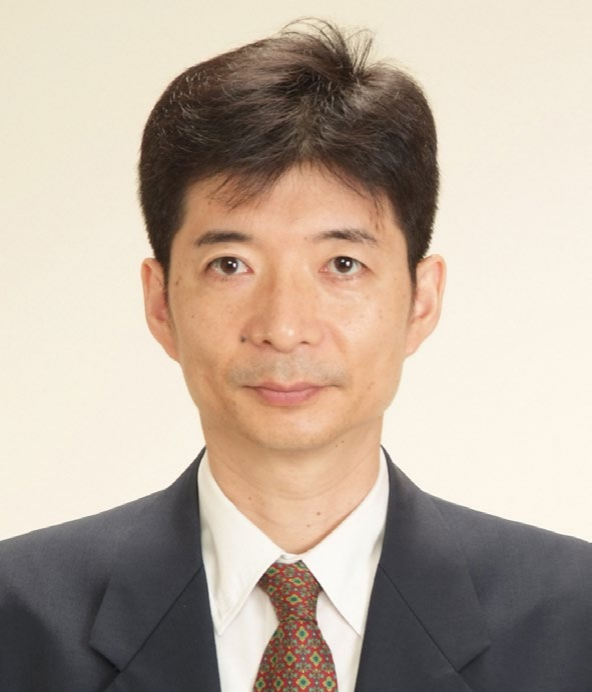

Pigmentary disorders encompass diverse hypopigmented and hyperpigmented disorders, affecting skin pigmentation and occasionally systemic organs. Individuals with pigmentary disorders may endure impaired quality of life and psychological burden. Recovery of health and wellness in suffered individuals could be achieved via elucidation of pathogenesis and innovation of novel therapeutic approaches with appropriate support. The most fruitful topics in pigmentary disorders in the recent decade are elucidation of pathogenesis in genetic pigmentary disorders, vitiligo, and chemical leukoderma (CL)/chemical‐induced vitiligo (CIV).

Okamura and Suzuki focus on genetic pigmentary disorders of hypopigmented disorders, hyperpigmented disorders, and RASopathies. They summarize recent novel findings in relationship between molecular mechanism and clinical manifestation. Okamura and Suzuki indicate that increasing genetic knowledge allows development of precision medicine approaches to personalized management strategies.

Inoue summarizes current knowledge on the factors leading to the onset and progression of vitiligo. Inoue integrates current facts about crosstalk between immune and non‐immune cells. Inoue presents the concept of “unstable equilibrium” between destructive and regulatory forces in the seemingly healthy non‐lesional skin in vitiligo patients. The concept might shift therapeutic strategies from treatment of vitiligo as a localized disorder to management of vitiligo as a systemic disease.

Kuroda, Yang and Katayama have investigated CL and CIV. They are currently revealing controversial processes in CL and CIV. Repigmentation and subsequent recovery occurs after eliminating the causative chemical in individuals with CL. A process of persistence, expansion, and/or appearance of other lesions occurs even after removing the causative chemical in persons with CIV. Kuroda, Yang & Katayama comment that numerous aspects of CIV pathogenesis remain to be clarified and that further research is required to facilitate the development of effective therapeutic interventions.

This special issue summarizes novel findings and would contribute to novel approaches for recovery of health and wellness in individuals with pigmentary disorders.

## Funding

The author has nothing to report.

## Conflicts of Interest

Naoki Oiso is an Editorial Board member of Journal of Dermatology and a co‐author of this article. To minimize bias, Naoki Oiso was excluded from all editorial decision‐making related to the acceptance of this article for publication.

## Data Availability

The data that support the findings of this study are openly available in naokioiso at https://doi.org/10.1111/1346‐8138.70157, reference number 20260108.

